# Exploration of the molecular characteristics and potential clinical significance of shared immune-related genes between preterm preeclampsia and term preeclampsia

**DOI:** 10.1186/s12884-024-06526-8

**Published:** 2024-08-15

**Authors:** Zhengrui Huang, Lu Sun, Yudie Gao, Meiting Shi, Ping Zhang, Yuzhen Ding, Jian Wang, Jiachun Wei, Xiuli Yang, Ruiman Li

**Affiliations:** 1 Department of Obstetrics and Gynecology, The First Affiliated Hospital of Jinan University, No. 613, Huangpu Road west, Tianhe district, Guangdong, 510630 China; 2 Department of Obstetrics and Gynecology, The Sixth Affiliated Hospital of Jinan University, No. 88, Changdong Road, Changping town, Dongguan, Guangdong 523000 China; 3 Department of Obstetrics and Gynecology, Peking University Shenzhen Hospital, Shenzhen, 518000 China

**Keywords:** Preterm preeclampsia, Term preeclampsia, Preterm birth, Immune signature, Nomogram, Immunotherapy

## Abstract

**Background:**

Preeclampsia is a severe obstetric disorder that significantly affects the maternal and neonatal peri-partum safety and long-term quality of life. However, there is limited research exploring the common mechanisms and potential clinical significance between early-onset preeclampsia and full-term preeclampsia from an immunological perspective.

**Methods:**

In this study, data analysis was conducted. Initially, immune-related co-expressed genes involving both subtypes of preeclampsia were identified through Weighted Gene Co-expression Network Analysis (WGCNA). Gene Ontology (GO) and Kyoto Encyclopedia of Genes and Genomes (KEGG) analyses were further employed to investigate the shared pathways regulated by immune-related genes. Binary logistic regression identified co-expressed genes with diagnostic value for preeclampsia, and a diagnostic model was constructed. Gene Set Enrichment Analysis (GSEA) predicted the potential biological functions of the selected genes. Lasso and Cox regression analyses identified genes closely associated with gestational duration, and a risk score model was established. A 4-gene feature, immune-related gene model for predicting the risk of preterm birth in preeclamptic pregnant women, was developed and validated through qPCR experiments. Immune cell infiltration analysis determined differences in immune cell infiltration between the two subtypes of preeclampsia.

**Results:**

This study identified 4 immune-related co-expressed genes (CXCR6, PIK3CB, IL1RAP, and OSMR). Additionally, diagnostic and preterm birth risk prediction models for preeclampsia were constructed based on these genes. GSEA analysis suggested the involvement of these genes in the regulation of galactose metabolism, notch signaling pathway, and RIG-I like receptor signaling pathway. Immune pathway analysis indicated that the activation of T cell co-inhibition could be a potential intervention target for immunotherapy in early-onset preeclampsia.

**Conclusion:**

Our study provides promising insights into immunotherapy and mechanistic research for preeclampsia, discovering novel diagnostic and intervention biomarkers, and offering personalized diagnostic tools for preeclampsia.

**Supplementary Information:**

The online version contains supplementary material available at 10.1186/s12884-024-06526-8.

## Introduction

Preeclampsia refers to a pregnancy-specific disorder that occurs after 20 weeks of gestation, characterized primarily by hypertension and proteinuria. According to statistics, it affects 4-5% of pregnancies worldwide, leading to the annual deaths of 60,000 pregnant women [1]. In addition to this, preeclampsia and its complications, such as preterm birth, fetal growth restriction, and damage to maternal end-organs, significantly impact the short-term and long-term quality of life for both mothers and newborns. Depending on the gestational age at onset, preeclampsia can be classified into preterm and term preeclampsia [1–3].

Preterm preeclampsia is associated with more severe adverse pregnancy outcomes and has attracted significant attention due to more pronounced pathological changes. Currently recognized diagnostic markers for preeclampsia, such as PLGF and sFLT-1, show better diagnostic value for preterm preeclampsia. From a pregnancy management perspective, the onset of term preeclampsia is not uncommon. Since the fetus is already mature at the time of onset, the adverse effects on the fetus are generally less compared to preterm preeclampsia [4]. However, from the perspective of long-term maternal and infant health, both subtypes of preeclampsia, once they occur, can induce widespread excessive maternal inflammatory responses and endothelial dysfunction. This increases the risk of postpartum hypertension in pregnant women. Genetic factors also contribute to an increased incidence of preeclampsia and hypertension in the offspring. In conclusion, the harm caused by preeclampsia to both mother and infant is not limited to the pregnancy period, emphasizing the need to explore more comprehensive biomarkers and disease intervention targets for preeclampsia [5, 6].

Preeclampsia is a heterogeneous placental-origin disease, where maternal vascular dysregulation, abnormal placental development, and overactive maternal-fetal interface and placental immune systems are considered the primary pathogenic mechanisms for preterm preeclampsia. Milder maternal vascular dysfunction and endothelial injury, along with an overactive placental immune system, are believed to be the main causes of term preeclampsia. By integrating the pathogenic mechanisms of both subtypes of preeclampsia, it can be observed that abnormal placental and syncytiotrophoblast cell functions are the main reasons for preeclampsia onset. Dysregulation of the placental immune system is a commonality in the pathological mechanisms of both subtypes of preeclampsia. An increasing number of researchers are also focusing on exploring potential intervention methods from an immunological perspective to enhance the current preeclampsia preventive treatment strategies, primarily centered around aspirin [7, 8].

Aspirin is currently widely used as a preventive treatment for preeclampsia, primarily acting through antiplatelet activity, inhibition of inflammation, protection of endothelial function, and reduction of the release of inflammatory factors, contributing to its therapeutic effects on preeclampsia [9]. However, from a pharmacological perspective, aspirin may increase the risk of bleeding and cause gastrointestinal reactions. From a clinical application standpoint, several clinical studies suggest that aspirin is most effective in preventing preeclampsia when taken between weeks 11–13 of pregnancy [10]. It is noteworthy that only a few developed countries and regions globally have the capacity to support high-quality early pregnancy preeclampsia diagnosis and aspirin preventive treatment strategies. Therefore, there is an urgent need to explore new diagnostic and intervention targets to further improve the existing treatment efficacy, expand the preventive treatment window for preeclampsia, and benefit a larger population.

The dynamic regulation of the immune environment in the placenta covers the entire pregnancy process, providing a longer intervention window in the temporal dimension. However, the placental barrier, while protecting the fetus, also hinders the entry of many large-molecule drugs, limiting their therapeutic efficacy [7, 8]. Gene therapy and small-molecule, highly lipophilic drugs hold promising prospects in placental immune therapy. Currently, researchers are exploring the use of PD-L1 to regulate T cells and modulate the biological functions of nourishing cells through binding with its receptors PD-L1 and PD-L2 for the treatment of preeclampsia [11, 12]. Tumor necrosis factor-α, interleukin-17, and the immune checkpoint Tim-3 have also captured the attention of researchers in the context of placental immune therapy [8, 13–15].

This study aims to explore commonalities in the pathogenic mechanisms of preterm preeclampsia and term preeclampsia from an immunological perspective by analyzing a placental dataset that includes data from both subtypes. The research identifies potential biomolecular targets with high diagnostic or intervention value. Preliminary validation of the analysis results is conducted using RT-PCR. Furthermore, a diagnostic model is constructed by integrating clinical information from the samples, and the potential diagnostic efficacy of the model for predicting preeclampsia and associated preterm delivery is evaluated.

## Materials and methods

### Dataset preparation

The dataset was obtained from the GEO database (https://www.ncbi.nlm.nih.gov/gds/), specifically GSE75010, which comprises 157 placental samples (basic sample information is provided in Table 1). From this dataset, we selected a total of 73 samples, including 31 term pregnancy and term preeclampsia placental samples (among them, 31 are from term pregnancies with preeclampsia, and 42 are from pregnancies of 37 weeks gestation or more), as well as 84 samples from preterm birth and preterm preeclampsia (comprising 49 samples from preterm preeclampsia and 35 samples from pregnancies terminated before 37 weeks). Furthermore, we obtained immune-related genes from the ImmPort database (https://www.immport.org), totaling 1793 genes (see Supplementary Table 1).

**Table 1:** Baseline characteristics of participants in dataset GSE75010

Characteristic	Preterm birth	Preterm preeclampsia	Term delivery	Term preeclampsia
Age	32.5 ± 5.6	32.8 ± 6.4	33.7 ± 5.4	33.8 ± 5.4
BMI	24.7 ± 5.0	28.2 ± 6.5	24.4 ± 5.1	24.2 ± 3.8
Gestational week	29.3 ± 2.5	29.9 ± 2.1	37.9 ± 1.5	37.16 ± 1.3
SBP	136.0 ± 24.3	173.7 ± 16.9	136.2 ± 22.71	163.6 ± 17.1
DBP	83.6 ± 14.8	109.6 ± 9.3	86.7 ± 14.2	103.7±
Infant sex (male/Female)	0/35	19/30	0/42	3/29
Previous pregnancy hypertension	1/35	8/49	3/42	8/31
HELLP symptom	0/35	8/49	5/42	8/31

BMI: Body mass index; SBP: systolic pressure; DBP: diastolic blood pressure; HELLP symptom: hemolysis, elevated liver enzymes and low platelets count syndrome

### Weighted gene co-expression network analysis (WGCNA)

**Fig. 1:**
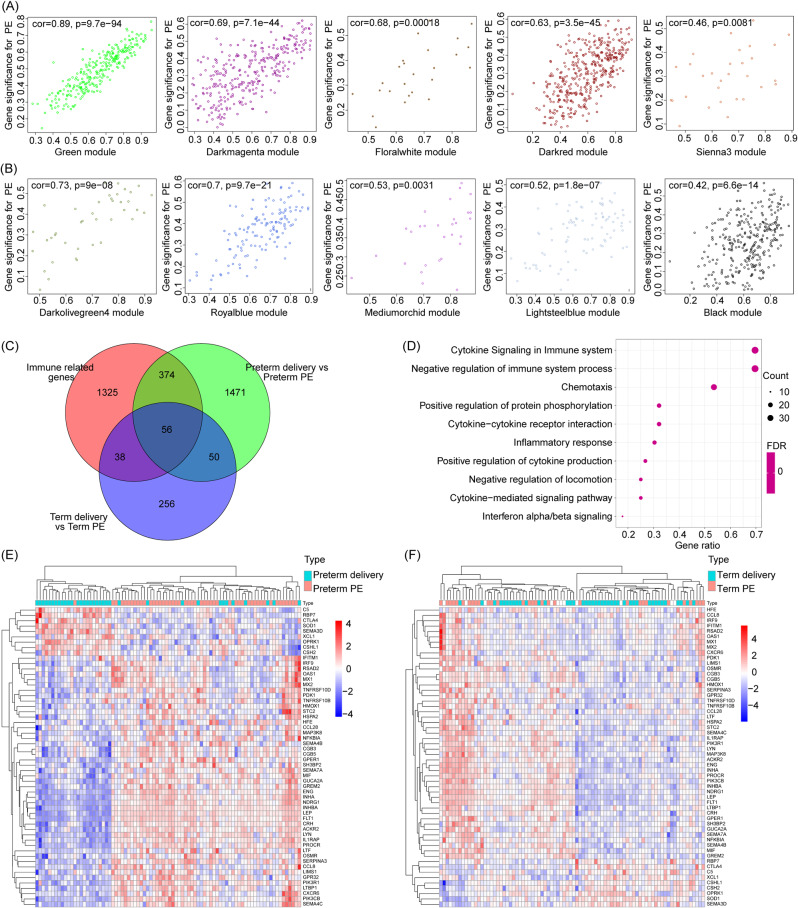
Gene significance scatter plot and identification of immune-related genes co-expressed in preterm preeclampsia and term preeclampsia. (**A**) Top five modules significantly associated with preterm preeclampsia. (**B**) The top five modules associated with term preeclampsia. (**C**) Shared genes overlapped between the first five modules of preterm preeclampsia, term preeclampsia and immune-related genes. (**D**) Top ten KEGG enrichment pathways of immune-related genes co-expressed in preterm preeclampsia and term preeclampsia. (**E**) Expression of immune-related co-expressed genes in preterm preeclampsia. (**F**) Expression of immune-related co-expressed genes in term preeclampsia

To mitigate the impact of gestational age on the analysis, we divided the samples into two independent matrices: one for preterm birth and preterm preeclampsia placenta, and the other for term pregnancy and term preeclampsia placenta. We conducted separate analyses on these two groups, selecting genes with expression differences of more than 50% for Weighted Gene Co-expression Network Analysis (WGCNA). Sample hierarchical clustering was performed using the “hclust” function in R. Subsequently, an unsigned network was constructed using the “WGCNA” R package, with R^2 soft thresholds set at 13 and 9 for the two groups, respectively. Genes with similar expression patterns were then clustered into the same modules, and highly similar gene modules were merged. Finally, Pearson correlation analysis was employed to assess the correlation between clinical features and gene modules. Genes within the top 5 modules ranked by correlation were extracted (Fig. 1A, B, where A represents the preterm preeclampsia group, and B represents the term preeclampsia group), and their intersection with immune-related genes obtained from ImmPort was identified (Fig. 1C). The expression profiles of these immune-related genes were visualized using a heatmap (Fig. 1E, F, where E represents the preterm preeclampsia group, and F represents the term preeclampsia group) [16, 17].

## GO and KEGG

The 56 immune-related genes obtained from the above analysis were subjected to functional analysis using the “clusterproflier” R package, and the top ten obtained KEGG pathways from the analysis were displayed using a bubble chart (Fig. 1D).

### Lasso and cox regression analysis

Firstly, we conducted preliminary screening of the 56 genes obtained from the above analysis using binary logistic regression, selecting genes with statistical significance for further analysis. Subsequently, we employed “Lasso” regression analysis for further refinement to enhance the model’s generalization ability.

The duration of gestational periods, the onset timing of the disease, the severity of the condition, and the occurrence of adverse pregnancy outcomes are closely related. In this study, we treated gestational age as survival data and utilized the “glmnet” R package to perform Cox regression analysis on the 56 immune-related genes for further variable selection. A significance level of *P* < 0.05 was considered statistically significant.

### GSEA analysis

To further investigate the role of the genes of interest in the pathogenesis of preeclampsia, we conducted potential regulatory pathway enrichment analysis based on the Kyoto Encyclopedia of Genes and Genomes (KEGG) using the ‘c2.cp.kegg.v7.2.symbols.gmt’ database. Selected genes were analyzed for enrichment in potential regulatory pathways. The genes within the gene set were ranked based on their expression values, and the Pearson correlation coefficients among all genes in the set were calculated. A threshold was set at NOM P-value < 0.05.

### Nomogram construction and evaluation

The cohort is divided into modeling and validation cohorts in a 7:3 ratio.

Based on the selected genes, we utilized the “rms” and “regplot” R packages to construct line plots for predicting preeclampsia and combined preeclampsia with preterm birth, respectively. Subsequently, the diagnostic performance of the candidate genes and the diagnostic model was assessed through Receiver Operating Characteristic (ROC) curves. Calibration testing and Decision Curve Analysis (DCA) were employed to evaluate the accuracy and clinical utility of the diagnostic model. Additionally, the diagnostic performance of the model was evaluated using ROC curves.

### Analysis of immune infiltration and immune activity among subgroups

In order to fully evaluate the changed immune environment of placenta, using cibersortx (https://cibersortx.stanford.edu/) online analytical software to analyze the placenta dataset GSE75010. Permutations for significance analysis were set to 1000. Analysis results of *P* < 0.05 were considered statistically significant. The expression results of 10 immune cells were displayed by multiple groups of bar charts, and the correlation between the proportion of interest genes and immune cells was analyzed. ssGSEA was performed by the “gsva” R package to calculate the activity of 13 immune-related pathways. The statistical method used was wilcoxon rank sum test.

### RT-PCR

Estimating sample size was performed using PASS (Power Analysis and Sample Size) software. According to recent studies, the worldwide incidence of preeclampsia is in the range of 2-4%. The allowable error (δ) was set to 0.05, and the estimated values of the overall rate (P) were set at 0.02 and 0.04, respectively. The formula N = zα/2 * P * (1 - P) / δ^2 was used for calculation. The confidence level was set at 0.95, with a confidence interval width of 0.05.

All placental specimens were used with permission from the Ethics Committee of the First Affiliated Hospital of Jinan University (Approval No.: KY-2021-054), and informed consent was obtained from the patients. Inclusion criteria for the normal group were the absence of any pregnancy complications, gestational age ≥ 37 weeks, and elective cesarean section due to the mother’s personal preference or a history of uterine scar. Inclusion criteria for the preterm group were termination of pregnancy due to cervical insufficiency or premature rupture of membranes. The diagnosis of preeclampsia was based on ACOG clinical guidelines, defined as new-onset hypertension (≥ 140/90 mmHg) after 20 weeks of gestation, accompanied by one or more of the following clinical features: proteinuria (≥ 300 mg/24 h), abnormal kidney, liver, or platelet function. Early-onset preeclampsia was defined if pregnancy termination occurred before 37 weeks based on the diagnosis of preeclampsia, while late-onset preeclampsia was defined if termination occurred at 37 weeks or later. Pregnant women with cardiovascular diseases, metabolic syndrome, immune system disorders, or liver and kidney diseases were excluded. To exclude the influence of confounding factors on placental immune status and gestational age, pregnant women with intrauterine growth retardation, premature rupture of membranes, amniotic fluid contamination, and intrauterine infection during pregnancy were not included in the study. Placental samples were collected immediately after delivery, and the sampling range was within 5 cm of the maternal surface of the placenta at the point of attachment to the umbilical cord. After washing with balanced salt solution to remove blood from the placental tissue, the samples were rapidly transferred to -80 °C for storage. Clinical baseline information for the specimens is displayed in Table 2.

**Table 2:** Baseline characteristics of participants in the study

Characteristic	Preterm birth (n = 21)	Preterm preeclampsia (n = 28)	Term delivery (n = 31)	Term preeclampsia (n = 23)
Age	32.5 ± 3.7	32.5 ± 5.2	32.6 ± 3.5	33.1 ± 4.4
BMI	25.3 ± 2.8	24.2 ± 3.5	26.7 ± 4.3	25.9 ± 4.7
Gestational week	34.6 ± 2.1	33.7 ± 2.9	38.3 ± 1.2	39.1 ± 1.7
SBP/DBP	122.4 ± 20.3	154.9 ± 12.3	117.6 ± 19.4	157.1 ± 23.5
	/73.3 ± 7.7	/87.4 ± 8.2	/82.3 ± 9.9	/90.3 ± 6.9
Smoking-yes	0/21	0/28	1/31	2/23
Infant sex (male/Female)	8/13	12/16	10/21	8/15
Previous pregnancy hypertension	1/21	3/28	0/31	2/23
HELLP symptom	0/21	3/28	0/31	1/23

BMI: Body mass index; SBP: systolic pressure; DBP: diastolic blood pressure; HELLP symptom: hemolysis, elevated liver enzymes and low platelets count syndrome

Initially, cell lysis buffer containing RNAase inhibitor was used for cell disruption, followed by tissue grinding at 4 °C. Total RNA was then extracted from the samples using the RNA simple total RNA extraction kit (DP419, TIANGEN, China), following the manufacturer’s instructions. In brief, the first step involved adding trizol reagent to lyse the cells and adding chloroform to extract RNA from cell debris. Isopropanol was used to precipitate RNA at room temperature for 20–30 min, followed by centrifugation at 4 °C at high speed to collect the RNA precipitate. The RNA precipitate was washed with 75% ethanol without RNAase, and after centrifugation to discard the supernatant, an appropriate amount of RNAase-free ddH2O was added to dissolve the precipitate. The concentration and purity of the extracted RNA were assessed using the OneDrop® OD-1000 nanodrop (OneDrop, OD-1000plus) Spectrophotometer. cDNA synthesis was carried out at 25 °C for 10 min, 42 °C for 15 min, and finally at 85 °C for 5 min using the Starscript II first-strand cDNA synthesis kit-II (A214-10, GenStar, Beijing). PCR was performed on the CFX ConnectTM Real-time PCR detection system (855,200, Bio-rad). The PCR primer sequences are provided in Table 3.

**Table 3:** PCR primer sequences

Gene name	Primer orientation	Sequence (5’-3’)
PIK3CB	Forward	TGCGACAGATGAGTGATG
	Reverse	CCTATCCTCCGATTACCAAG
IL1RAP	Forward	CAAGGAATGCGGGAAGAAGA
	Reverse	GGCTTAGAACAACCAGGAG
CXCR6	Forward	GGAGGAGCATCAAGACTTC
	Reverse	GATATGACCAGCACCAGAG
OSMR	Forward	GAGTGAAGTCTTGGCTGAA
	Reverse	AGGAAGGTTGTGGACAGT
GAPDH	Forward	TATGACAACAGCCTCAAGAT
	Reverse	AGTCCTTCCACGATACCA

### Statistical analysis

Statistical analysis was expressed as mean ± SD, and SPSS 26 software was used for data analysis. Through testing, the data in this experiment do not have homogeneity of variance and do not obey normal distribution. Therefore, the experimental data were analyzed using the wilcoxon rank sum test comparing two independent samples. *P* < 0.05*, *P* < 0.01**, *P* < 0.001*** (see Fig. 2).

## Results

**Fig. 2:**
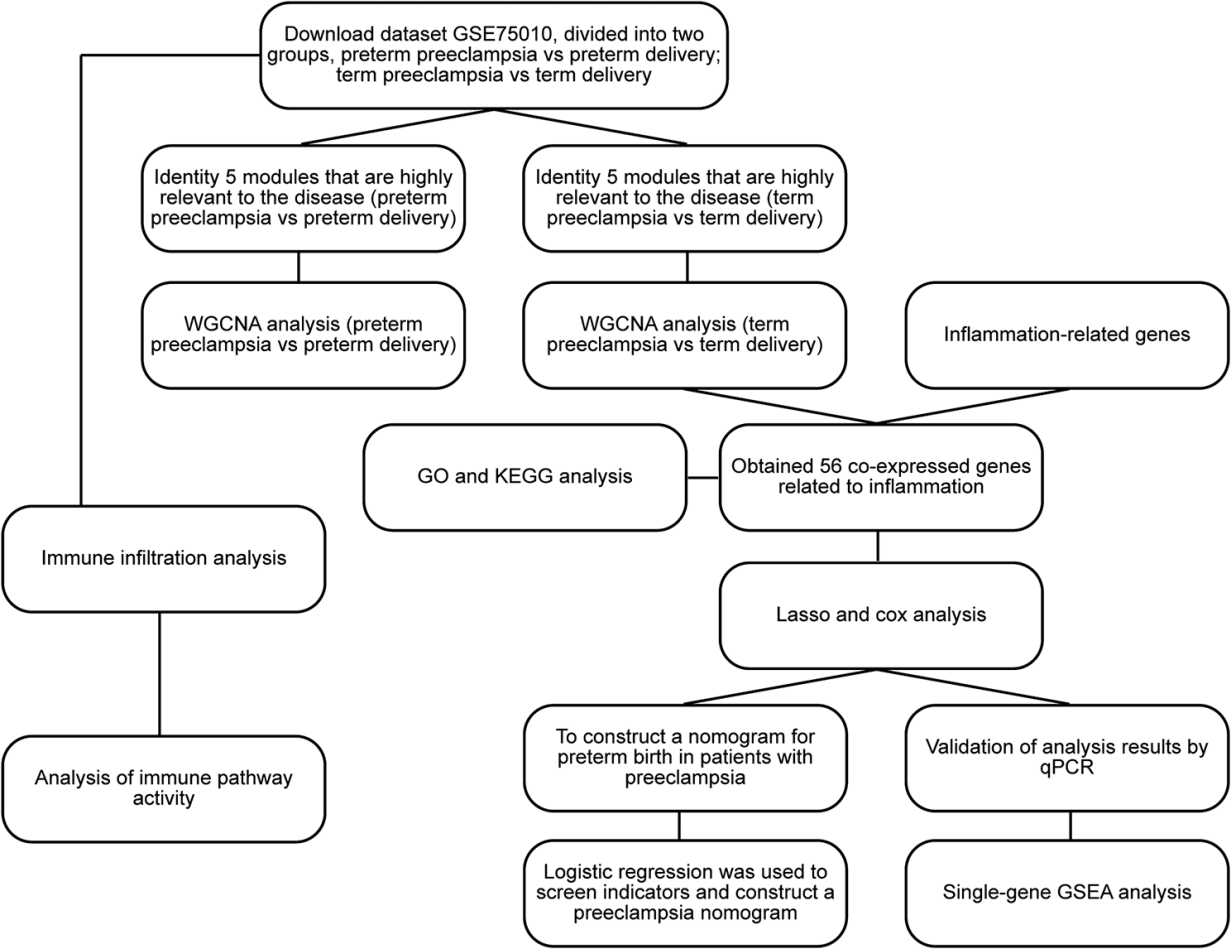
Depicts the study flow chart

### Identification of immune-related coexpression gene

**Fig. 3:**
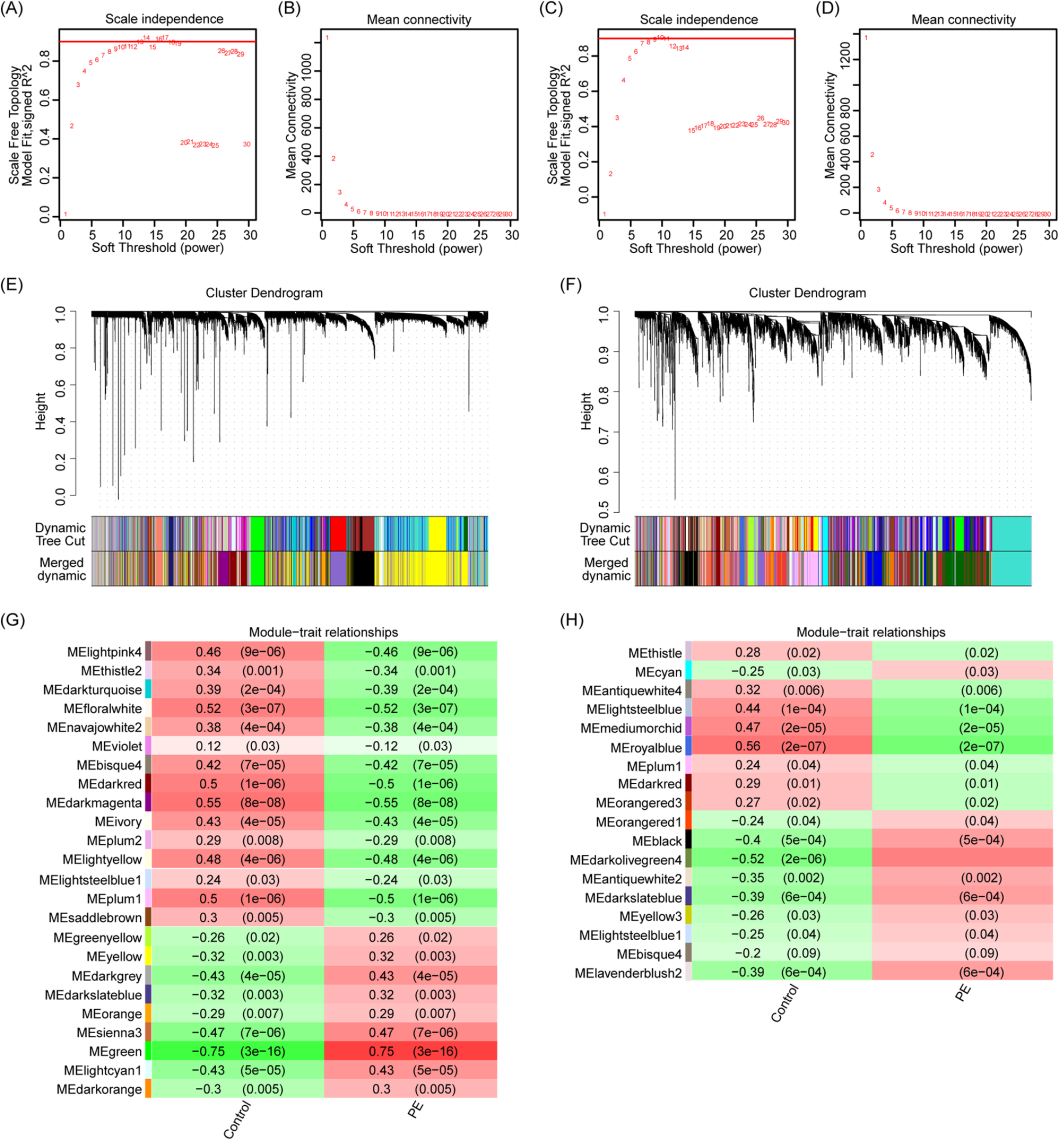
Weighted gene co-expression network analysis (WGCNA). (**A**, **B**) Determine the weighted value β that satisfies the scale-free network law in the matrix about preterm preeclampsia. (**C**, **D**) Determine the weighted value β that satisfies the scale-free network law in the matrix about term preeclampsia. (**E**) The original and merged modules under the cluster tree in the preterm preeclampsia matrix. (**F**) The original and merged modules under the cluster tree in the term preeclampsia matrix. (**G**) Module-trait correlation heat map of preterm preeclampsia. (**H**) Module-trait correlation heat map of term preeclampsia

In order to identify co-expressed genes strongly associated with early-onset preeclampsia and term preeclampsia, Weighted Gene Co-expression Network Analysis (WGCNA) was conducted (the details of soft threshold setting and matrix construction are illustrated in Fig. 3A-D, where A-B corresponds to the preterm preeclampsia group, and C-D corresponds to the term preeclampsia group. The cluster dendrogram is presented in Fig. 3E, F, with E representing the preterm preeclampsia group, and F representing the term preeclampsia group. Module-trait relationships are depicted in Fig. 3G, H, where G represents the preterm preeclampsia group, and H represents the term preeclampsia group). Analysis of the matrix combining preterm and preterm preeclampsia groups revealed the top five modules most strongly correlated with early-onset preeclampsia as MEgreen (cor = 0.89, *P* < 0.01), MEsienna3 (cor = 0.46, *P* < 0.01), MEyellow (cor = 0.39, *P* < 0.01), MEdarkgrey (cor = 0.63, *P* < 0.01), and MEbrown4 (cor = 0.63, *P* < 0.01) (genes within each module are presented in Supplementary Table 2). Similarly, the top five modules most strongly correlated with term preeclampsia were identified as MEdarkolivegreen4 (cor = 0.73, *P* < 0.01), MEantiquewhite2 (cor = 0.42, *P* < 0.01), and Medarkslateblue (cor = 0.27, *P* < 0.01) (genes within each module are presented in Supplementary Table 3).

The intersection of highly correlated genes with early-onset preeclampsia, highly correlated genes with term preeclampsia, and immune-related genes obtained from ImmPort resulted in 56 immune-related genes associated with both early-onset and term preeclampsia (Fig. 3C). The list of these 56 genes is presented in Supplementary Table 3.

### GO and KEGG analysis

GO analysis showed that the top six biological processes enriched in 56 immune-related genes were signal transduction, inflammatory response, positive regulation of cell migration, response to virus, negative regulation of viral genome replication and chemotaxis; the top five cellular components are plasma membrane, extracellular space, extracellular region, integral component of plasma membrane, perinuclear region of cytoplasm; the top three enriched molecular functions are hormone activity, chemorepellent activity, semaphorin receptor (Supplementary Fig. 1). KEGG analysis indicated that the top ten enriched KEGG terms were cytokine signaling in immune system, negative regulation of immune system process, chemotaxis, positive regulation of protein phosphorylation, cytokine-cytokine receptor interaction, inflammatory response, positive regulation of cytokine production, negative regulation of locomotion, cytokine-mediated signaling pathway and interferon alpha/beta signaling (Fig. 3D).

### Construction of immune gene-related prognostic model

**Fig. 4:**
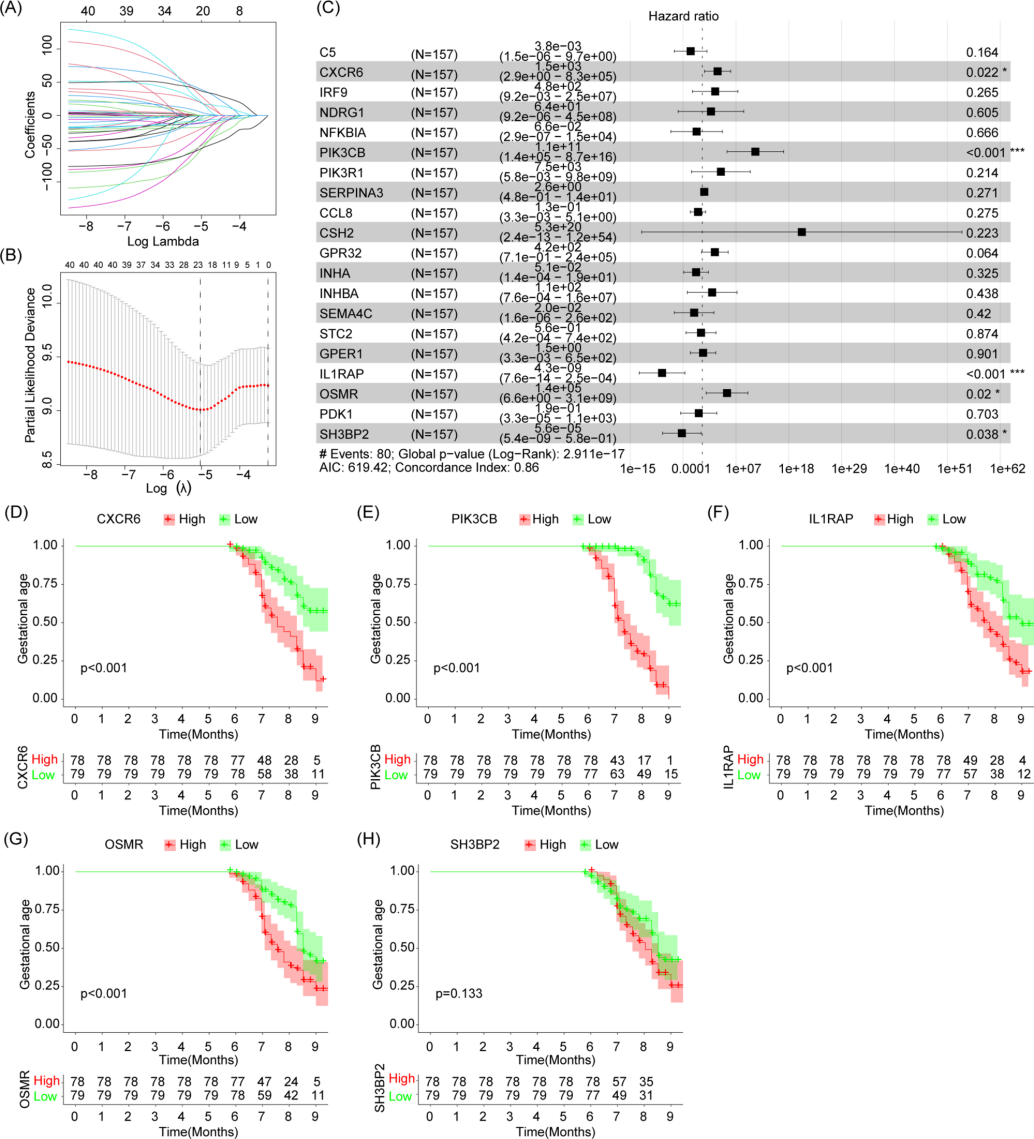
Diagnostic efficacy of selected genes for gestational age. (**A**) Lasso coefficient curve. (**B**) Based on the lowest criteria of OS, the adjustment parameter (lambda) in the lasso model was selected. (**C**) Multivariate cox regression analysis forest plot of selected immune-related genes based on Lasso regression analysis. (**D**–**H**) Kaplan-Meier curves for 5-immune-related genes to evaluate the effect of 5-immune-related genes on the gestational age

The effect of 56 immune-related genes on gestational weeks was assessed based on clinical information from dataset GSE75010. First, 20 immune-related genes related to gestational age were identified by lasso analysis, NDRG1, IRF9, SERPINA3, CXCR6, NFKBIA, PIK3R1, PIK3CB, C5, SEMA4C, GPR32, CRH, CSH2, INHA, INHBA, STC2, GPER1, IL1RAP, OSMR, SH3BP2, PDK1. Multivariate cox regression analysis was used to further screen variables from the above 20 immune genes associated with gestational age, and finally five immune-related genes with statistical significance (IL1RAP, PIK3CB, OSMR, SH3BP2 and CXCR6) were included for further evaluation (Fig. 4A-C).

Kaplan-Meier survival analysis was used to evaluate the diagnostic value of the above five selected genes for gestational age. The analysis results showed that IL1RAP, PIK3CB, OSMR and CXCR6 had good discrimination ability for gestational age and were statistically significant (Fig. 4D-H).

**Fig. 5:**
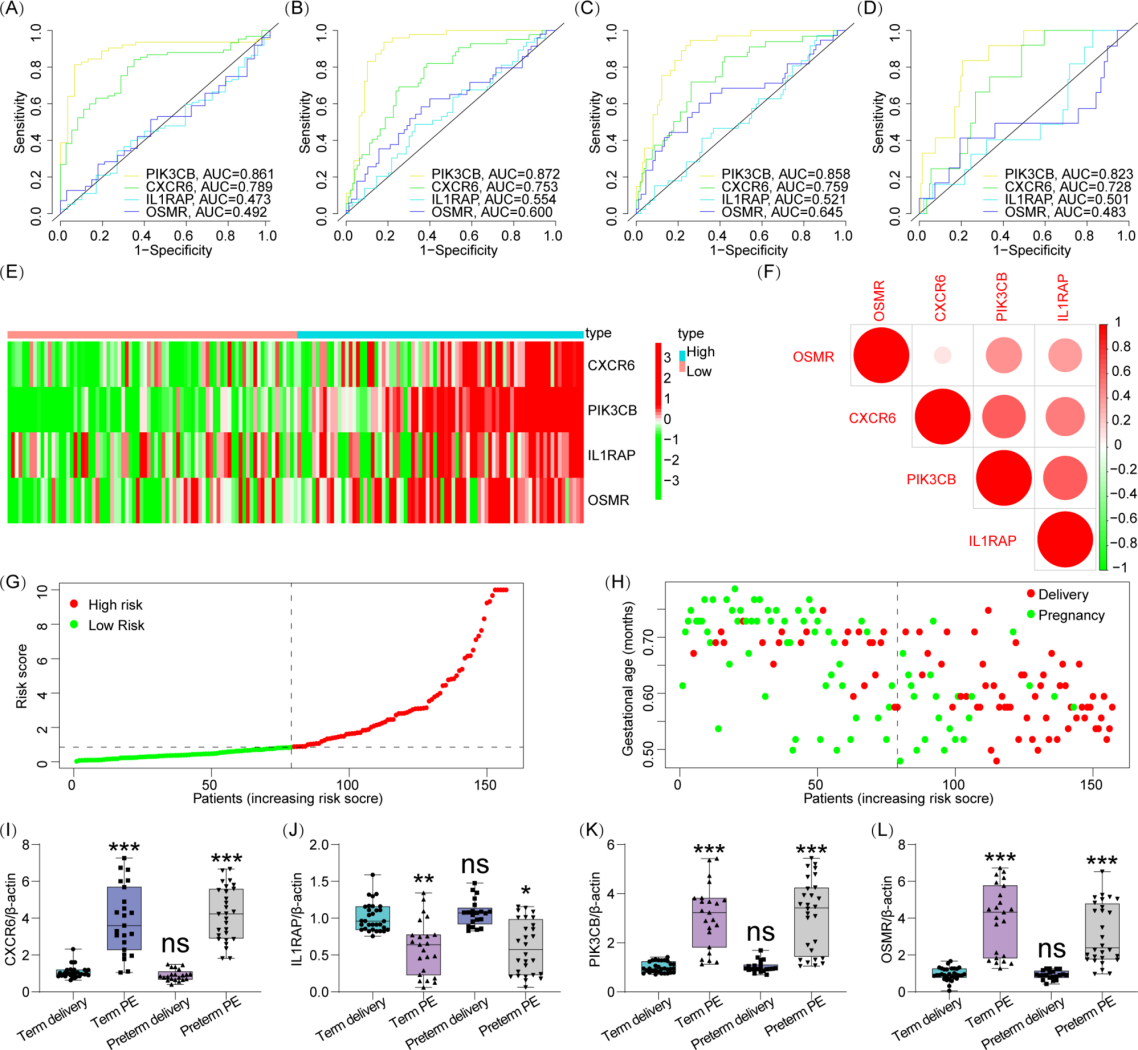
Risk assessment model. (**A**–**D**) The time-dependent ROC curve was used to evaluate the sensitivity and specificity of the selected genes for predicting preterm birth < 37 weeks, <34 weeks, <32 weeks and <28 weeks. (**E**) Expression patterns of 4-immune-related genes in high and low risk populations. (**F**) Correlation analysis among four selected genes. (**G, H**) Ranking points and scatter plots of the distribution by risk score. (**I**–**L**) Expression of selected genes at the mRNA level

Time-dependent ROC analysis showed that PIK3CB alone had the best diagnostic value for discriminating gestational age (< 37 weeks was AUC = 0.861, < 34 weeks was AUC = 0.872; < 32 weeks was AUC = 0.858, < 28 weeks was AUC = 0.823) (Fig. 5A-D, where A represents the diagnosis value of each genes < 28 weeks, B represents the diagnosis value of each genes < 32 weeks, C represents the diagnosis value of each genes < 34 weeks, and D represents the diagnosis value of each genes < 37weeks).

Patients were divided into high-risk and low-risk groups based on the median gestational age. Figure 5E illustrates the expression of each gene in the high-risk and low-risk groups in the preterm birth risk model. Figure 5F shows the correlation between the four selected genes. The preterm birth risk for each pregnant woman was estimated according to the fitted risk assessment model, and the scores and risk assessment results for each pregnant woman are shown in Fig. 5G, H.

### RT-PCR

Combined with the incidence rate of preeclampsia, the results of sample estimation suggest that a convincing sample size of 50–54 samples per disease group is needed. Therefore, we collected 52 control group placental tissues, including 21 from preterm births and 31 from full-term pregnancies. Additionally, we obtained 51 preeclampsia group placental tissues, comprising 28 from preterm preeclampsia and 23 from full-term pregnancies with preeclampsia. PCR experiment results indicate an increase in the expression levels of CXCR6, PIK3CB, and OSMR at the RNA level in both subtypes of preeclampsia. Furthermore, the expression of IL1RAP in placental tissues of preeclampsia patients was found to be decreased (Fig. 5I-L).

### GSEA

**Fig. 6:**
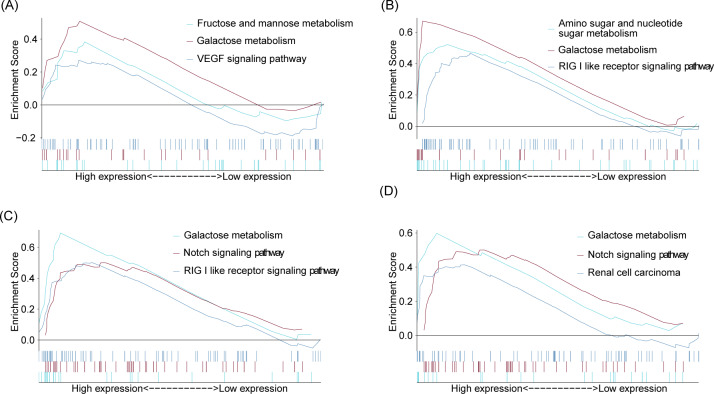
GSEA analysis results. (**A**) The top three enriched terms in the GSEA analysis for IL1RAP. (**B**) The top three enriched terms in the GSEA analysis for PIK3CB. (**C**) The top three enriched terms in the GSEA analysis for OSMR. (**D**) The top three enriched terms in the GSEA analysis for CXCR6

GSEA analysis results suggested that the first three enrichment pathways of IL1RAP were VEGF signaling pathway, fructose and mannose metabilism and galactose metabolism (Fig. 6A). The first three enrichment pathways of PIK3CB are galactose metabolism, RIG I like receptor signaling pathway and amino sugar and nucleotide sugar metabolism (Fig. 6B); The top three enrichment pathways of OSMR are notch signaling pathway, galactose metabolism and RIG I like receptor signaling pathway (Fig. 6C); The first three enrichment pathways of CXCR6 are renal cell carcinoma, galactose metabolism, and notch signaling pathway (Fig. 6D).

Synthesize the above analysis results, the results of GSEA analysis indicated that five genes of interest were mainly involved in the regulation of galactose metabolism, notch signaling pathway and RIG I like receptor signaling pathway (Fig. 6).

### Nomogram construction

**Fig. 7:**
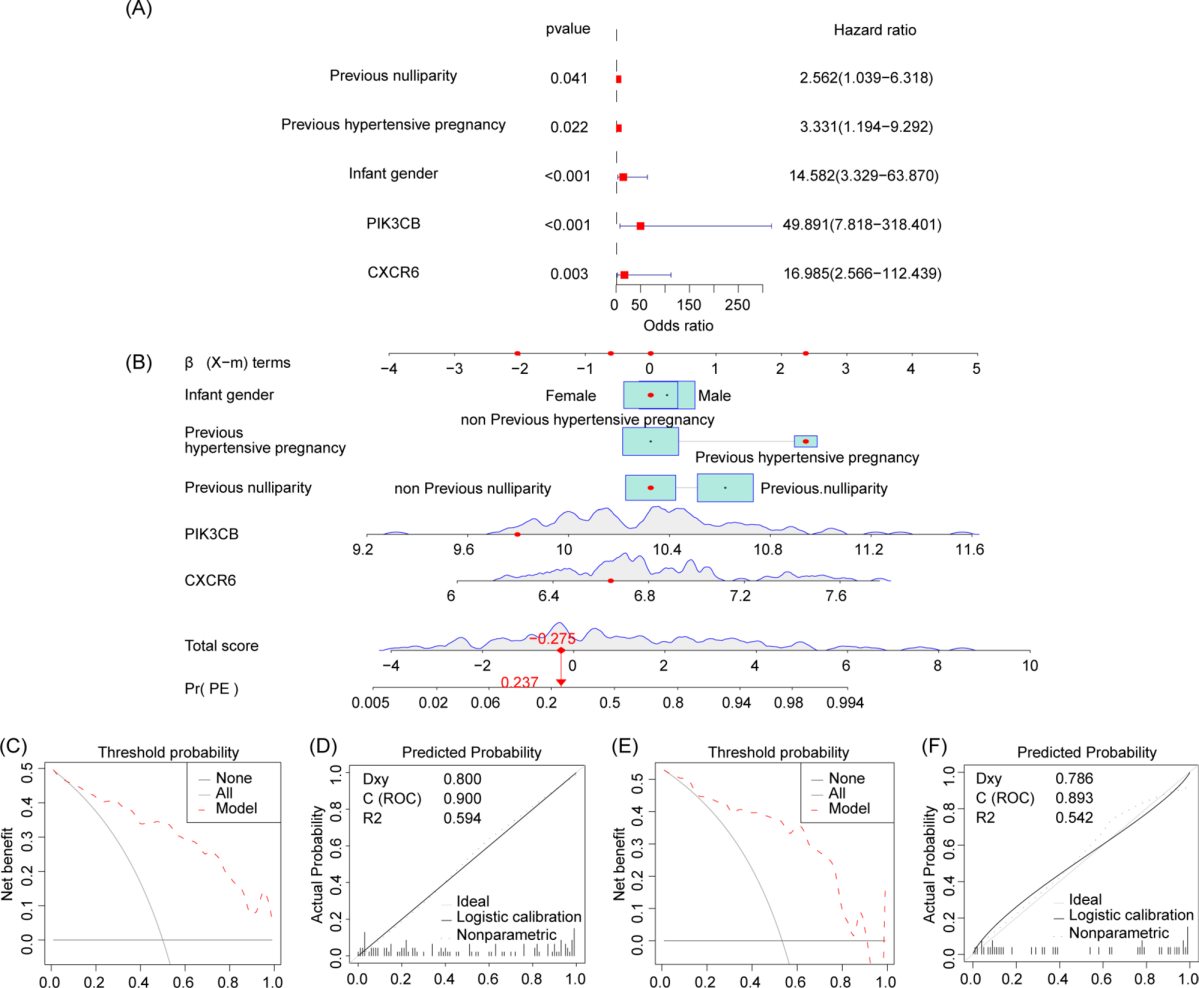
Construction of a diagnostic nomogram for preeclampsia. (**A**) Logistics regression analysis was used to screen indicators with diagnostic value. (**B**) Diagnostic nomogram for preeclampsia. (**C**, **D**) Calibration test and DCA analysis of modeling cohort. (**E**, **F**) Calibration test and DCA analysis of the validation cohort

First, we used logistics analysis to identify clinical information and biomarkers with diagnostic value for preeclampsia. The results showed that previous nulliparity, previous hypertension pregnancy, infant gender, PIK3CB and CXCR6 has diagnostic value for preeclampsia (Fig. 7A), so a preeclampsia nomogram combined with the above indicators was constructed (Fig. 7B). Calibration test and DCA analysis showed that the nomogram has high accuracy and net benefit in both modeling cohort (Fig. 7C, D) and validation cohort (Fig. 7E, F).

**Fig. 8:**
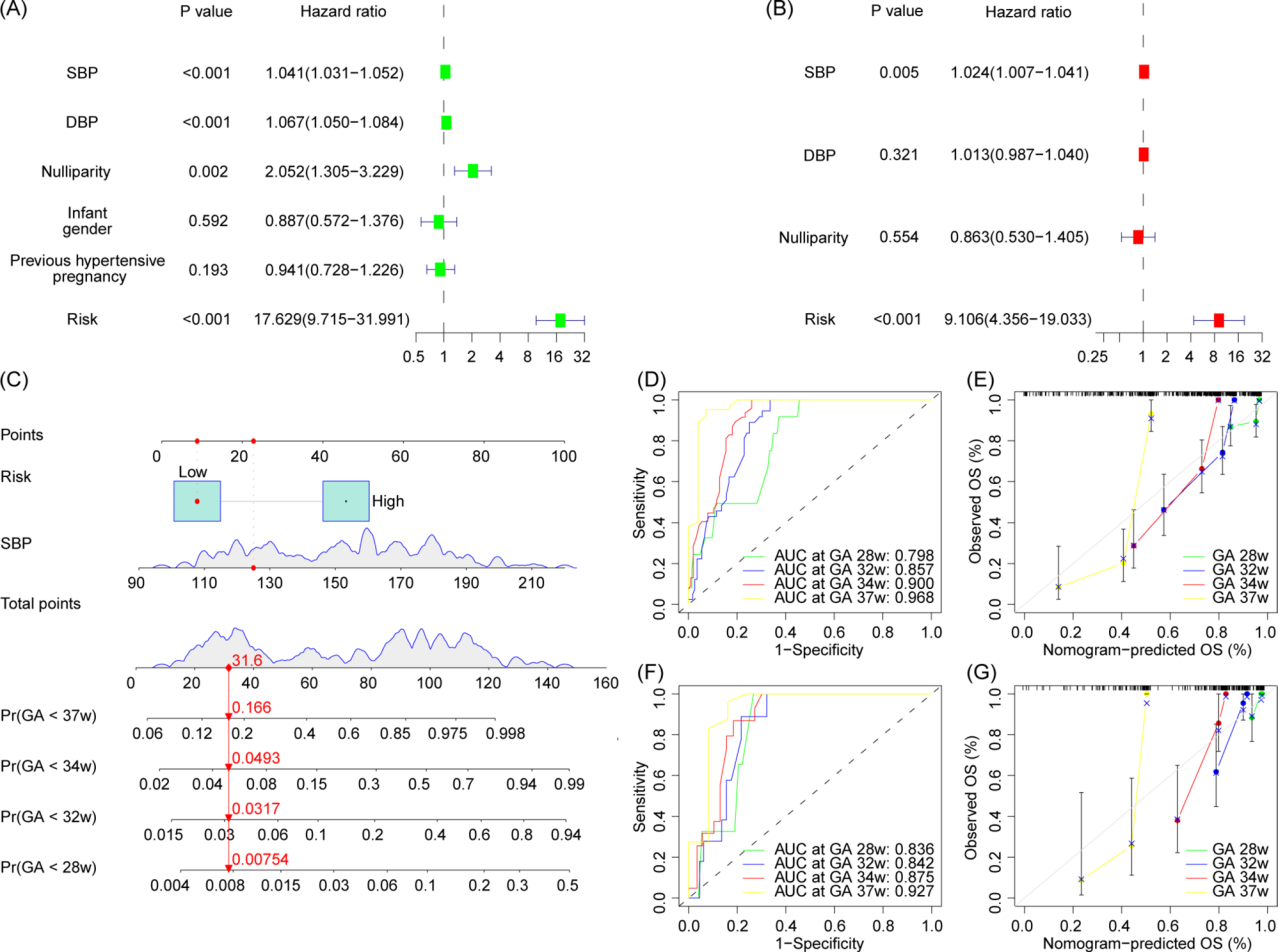
Construction of a nomogram for preeclampsia complicated with preterm birth. (**A**) Univariate cox regression analysis. (**B**) Multivariate cox regression analysis to select variables included in the nomogram. (**C**) The nomogram of preeclampsia complicated with preterm birth. (**D**, **E**) Calibration test and time-dependent ROC curve of modeling cohort. (**F**, **G**) Calibration test and time-dependent ROC curve of validation cohort

Short gestational age seriously endanger fetal development and safety, and it is also an indicator of the situation of the disease. Therefore, we constructed a nomogram to predict the delivery time and the whole gestational age of patients with preeclampsia, and to provide guidance for the active prevention and treatment of PE and the extension of pregnancy cycle. Based on the above Kaplan-Meier survival analysis and time-dependent ROC analysis results, we used risk model mentioned to construct a nomogram to predict the risk of preterm birth < 28 weeks, < 32 weeks, < 34 weeks and < 37 weeks in preeclampsia patients (Fig. 8C). Univariate and multivariate cox regression analysis suggested that systolic blood pressure and risk model had better diagnostic efficacy in preeclampsia combined with preterm birth, so the above indexes were included in the nomogram (Fig. 8A, B). Calibration test and time-dependent ROC analysis were used to evaluate the accuracy and efficacy of the nomogram, and the results showed that the nomogram had good performance at all four time points (Fig. 8D, E for modeling cohort; Fig. 8F, G for validation cohort).

### Immune infiltration analysis

**Fig. 9:**
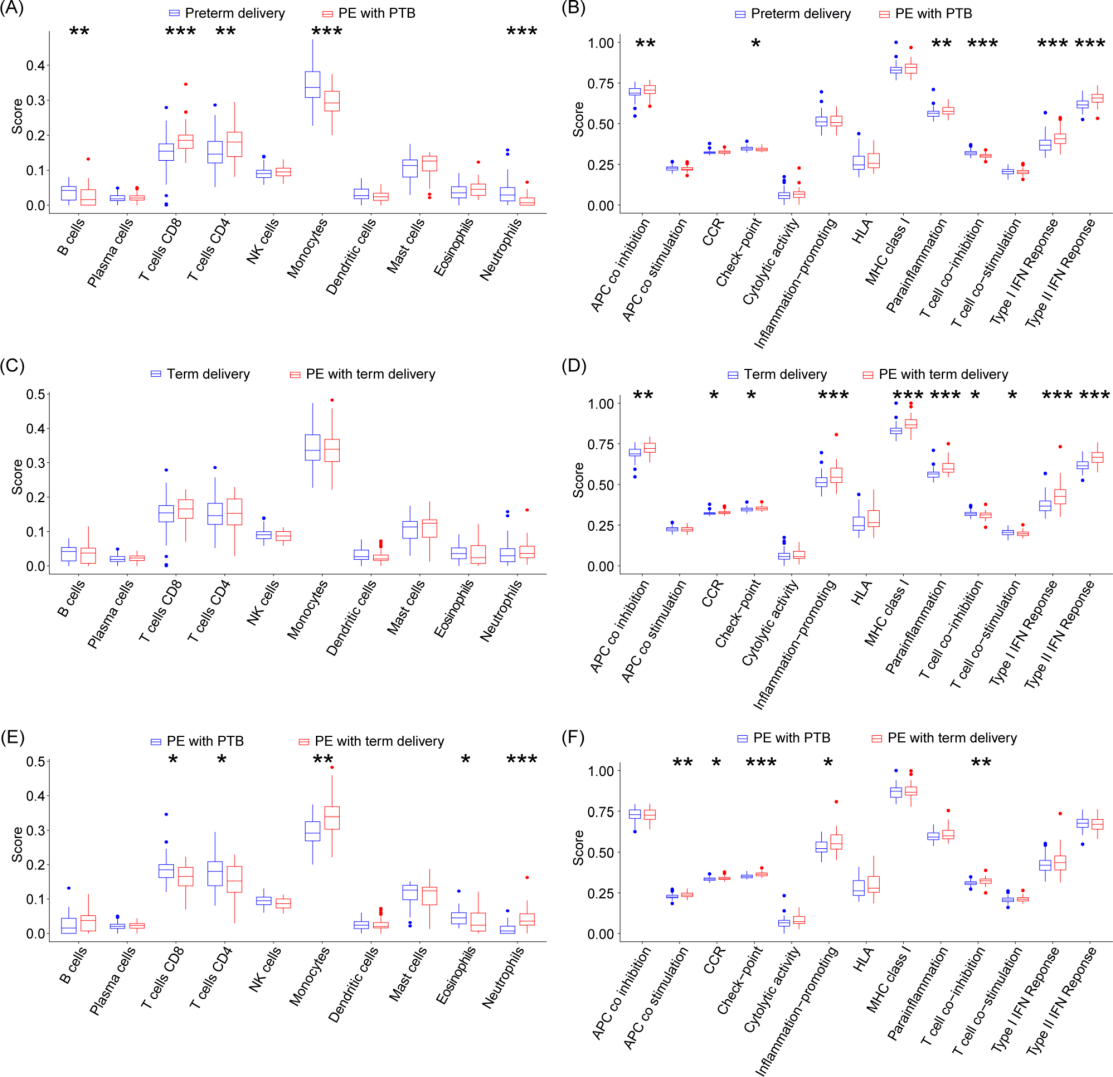
Immunoinfiltration and immunopathway activity analysis. (**A**, **B**) Immune infiltration and immune pathway activity in preeclampsia preterm. (**C**, **D**) Immune infiltration and immune pathway activity in term pregnancy-induced preeclampsia. (**E**, **F**) To compare placental immune infiltration and immune pathway activity in preeclampsia of preterm pregnancy and preeclampsia of term pregnancy

To further understand the changes of placental immune environment in preterm preeclampsia and term preeclampsia and to explore the immune-related pathophysiological mechanisms of preeclampsia subtypes, immune cell infiltration and immune function analysis were performed. The results of comparison between preterm preeclampsia and preterm delivery group suggested that B cells, monocytes and neutrophils decreased in preterm preeclampsia and were statistically significant, the number of T cell CD8 and T cell CD4 was increased (Fig. 9A). The comparison results suggests that term preeclampsia has a similar trend as that between term preeclampsia and term delivery group, but did not have statistical significance (Fig. 9C); The comparison of preterm preeclampsia and term preeclampsia suggested that T cell CD8, T cell CD4, and eosinophils increased significantly in the placenta of preterm preeclampsia; monocytes, neutrophils were significantly increased in the placenta of term preeclampsia (Fig. 9E).

In preterm preeclampsia versus the preterm delivery group, APC-co-inhibition, MHC class I, parainflammatation, type I IFN response and type II IFN response is upregulated in preterm preeclampsia, whereas check point and T cell co-inhibition pathway are down-regulated in preterm preeclampsia (Fig. 9B). In term preeclampsia versus the term delivery group APC-co-inhibition, CCR, check point, HLA, MHC class I, parainflammatation, type I IFN response and type II IFN response is upregulated in term preeclampsia; T cell co-inhibition, T cell co-stimulation is down-regulated in term preeclampsia (Fig. 9D). The comparison of preterm preeclampsia with term preeclampsia shows that APC-co-stimulation, CCR, check point, HLA and T cell co-inhibition are upregulated in the placenta of term preeclampsia (Fig. 9F).

## Discussion

The imbalance in the immune environment during pregnancy serves as the root cause for a spectrum of obstetric diseases, with preeclampsia being one of the more severe conditions. The “two-stage” model is currently widely accepted as the pathogenesis of preeclampsia. The first stage, known as the clinical antecedent phase, involves various factors leading to placental ischemia, hypoxia, and stress-induced activation of syncytiotrophoblasts. During this stage, the primary site of immune dysregulation occurs at the maternal-fetal interface. In the second stage, an excessive inflammatory response in the placenta exacerbates the progression of the disease [18, 19]. The appearance of stress-induced activation in syncytiotrophoblasts and the subsequent placental functional disruptions, including immune environment dysregulation, collectively constitute a shared pathological outcome for all subtypes of preeclampsia. This study, through the analysis of a placental dataset, identified inflammation-related genes such as CXCR6, PIK3CB, IL1RAP, and OSMR coexisting in early-onset and full-term preeclampsia. Preliminary validation through PCR was conducted to confirm the analysis results, leading to the development of diagnostic models for preeclampsia and preterm birth based on the selected genes. A comparison was made between the immune infiltration and activity changes in immune-related pathways for preterm and term preeclampsia.

The results of GO and KEGG analyses indicate that 56 co-expressed genes are extensively involved in the regulation of the immune environment in placental tissue. These genes primarily exert a negative regulation on the immune system and leukocyte activity, possibly leading to changes triggered by the suppression of excessive immune responses. The negative regulation of chemotaxis inhibits the aggregation of immune cells. In addition to the regulation of the immune system, the co-expressed genes inhibit the secretion of gonadotropins, which may result in the suppression of placental growth-related factors and the secretion of placental hormones. This inhibition could also potentially slow placental development and lead to insufficient oxygen and nutrient supply to the fetus. This study identified four immune-related genes, namely CXCR6, PIK3CB, and OSMR, whose roles in the pathogenesis of preeclampsia have not been fully elucidated. CXCR6 is a member of the CXC chemokine receptor family, and CXCL16 is its ligand with high specificity. Current studies suggest that CXCL16/CXCR6 are mainly involved in the recruitment of decidual T lymphocytes and monocytes, promote endometrial decidualization under pregnancy conditions [20–23]. Activation of PIK3CB involves multiple signaling cascades of cell growth, survival, proliferation, etc [24, 25], which have only been mentioned in a few transcriptomic studies in gynecology and obstetrics related diseases and lack of in-depth mechanism studies [26–28]. Currently, IL1RAP is believed to be involved in the pathogenesis of preeclampsia by inhibiting trophoblast proliferation, migration, invasion, and angiogenesis [29–31]. OSMR is produced mainly by white blood cells, is a member of the interleukin-6 receptor family and binds to interleukin-31. OSM/OSMR signaling plays an important role in inflammation, hematopoiesis, development, and is increasingly recognized as an important factor in cancer progression, but is still underrepresented in perinatal disease studies [32, 33]. In summary, except for a few studies on the role of IL1RAP in trophoblast, the role of the other four genes in the pathogenesis of preeclampsia is still worth further investigation.

This study employed Gene Set Enrichment Analysis (GSEA) to predict the roles of the aforementioned genes in the pathogenesis of preeclampsia. The analysis results suggest that these genes are primarily involved in the regulation of galactose metabolism, the notch signaling pathway, and the RIG-I-like receptor signaling pathway. Our analysis specifically indicates the involvement of CXCR6, PIK3CB, and OSMR in the regulation of the RIG-I-like receptor signaling pathway, a common immune signaling pathway crucial for innate immunity, inflammation, and the upregulation of antiviral proteins to control viral infections [34, 35]. Simultaneously, our research identifies IL1RAP, CXCR6, PIK3CB, and OSMR as being enriched in the galactose metabolism pathway. Lactose is an essential carbohydrate in cellular metabolism, serving as a precursor for glycosylation and participating extensively in the regulation of lipid and protein functions, intercellular communication, immune functions, and intracellular signal transduction. Notably, CXCR6 and OSMR also participate in the regulation of the amino sugar and nucleotide sugar metabolism pathway. The role of energy metabolism is increasingly recognized in the development of various diseases and the regulation of the body’s immune functions. In the pathogenesis of preeclampsia, the insufficient energy production by nourishing cells contributes to their inability to sustain rapid proliferation and normal functionality, further exacerbating the deterioration of the pregnancy environment [36–39]. Among the four identified genes of interest, aside from the study by Xiaomin Zhao et al. in 2018, which revealed the role of micro-4331 in promoting mitochondria damage induced by gastrointestinal viruses through upregulating IL1RAP, further research is needed to elucidate the impact of CXCR6, PIK3CB, and OSMR on cellular energy metabolism and mitochondrial function.

Our study showed that due to the changes of immune cell subtypes and numbers, such as the increase of different B cells, T cells, monocytes, and the decrease of neutrophils. Placentas with preterm preeclampsia may have more intense and complex changes in the immune environment than those with term preeclampsia. In addition, our study found that immunocheckpoints APC-co-inhibition, parainflammatation, type I IFN response and type II IFN response have the same overexpression trends in both subtypes of preeclampsia, and T cell co-inhibition has the same downward trend. APC(Antigen-presenting cells) is a major participant in adaptive immune responses. Previous studies suggest that the number of antigen-presenting cells in the placenta of preeclampsia women is increased, but our study suggests increased activity of APc-co-inhibition pathway, this doubt still needs further study to prove. In addition flammation is graded and can be divided into homeostasis, inflammation, and parainflammation in the middle. Unlike the other two extremes, “parainflammation” contributes to the adaptation of the tissue to harmful conditions and the restoration of flammation and depends on the aid of resident macrophages, our findings highlight the existence of a paraninflammatory state in the placental tissue of preeclampsia and the potential of macrophages as a novel target for the treatment of preeclampsia. Interferon regulatory factors (IFN) are a family of transcription factors, and the effects of the IFN family on the immune environment during pregnancy have been widely studied. This study suggests two potential intervention targets for type I IFN response and type II IFN response. Immunocheckpoint T cell co-inhibition also can be a potential intervention target for inhibiting excessive T cell activity in preeclampsia placenta [8].

The paragraph discusses the construction of a line chart to evaluate the potential value of selected genes in the diagnosis and management of preeclampsia. Binary logistic regression analysis suggests that PIK3CB and CXCR6 have good diagnostic value for preeclampsia. The diagnostic model constructed with regression coefficients demonstrates excellent diagnostic performance (modeling dataset AUC = 0.900, validation dataset AUC = 0.893). Calibration tests indicate the model’s accuracy, and clinical decision curve analysis suggests good clinical diagnostic benefits. In addition to predicting preeclampsia, controlling the timing of delivery is crucial. Premature termination of pregnancy limits fetal respiratory and nervous system development. Pregnancy duration is influenced by the severity of the disease and maternal tolerance. Prolonging pregnancy provides more growth and development time for the fetus but may cause greater harm to the mother. Balancing maternal tolerance and fetal development is a key factor in deciding to terminate pregnancy. Therefore, combining four selected genes, we construct a preterm birth risk model and further develop a diagnostic model for preeclampsia combined with preterm birth. The results suggest that the model has good diagnostic value for preeclampsia combined with preterm birth. However, it is acknowledged that the preterm birth risk model is solely based on maternal factors, and further research is needed to assess the predictive value of these genes in fetal development.

We are keenly aware of the limitations in our study. Firstly, as an increasing number of omics technologies are employed to explore the pathogenesis of diseases, our research is based solely on placental transcriptomics and has not adequately integrated metabolomics, genomics, epigenetics, and other technologies to provide a more comprehensive description of the onset and development of the disease. Secondly, in the exploration of clinical applications, due to the limited sample size of the dataset, we could only preliminarily assess the potential diagnostic value of biomarkers, and further clinical studies are needed for confirmation. Additionally, as preeclampsia is a dynamically evolving disease, there is a need to develop dynamic disease diagnostic models based on clinical validation to enhance diagnostic efficiency in assessing the disease’s status. At the experimental design level, we made efforts to minimize bias caused by variations in gestational weeks and selected placentas from preterm patients with non-placental insufficiency or immune-related factors. However, due to the temporal constraints of human specimen collection, our study can only suggest a certain correlation between the abnormal expression of selected genes and the presence of preeclampsia, making it challenging to exclude the impact of labor on gene expression. Further animal experiments are still required to elucidate the roles these genes play in the development of preeclampsia and throughout the entire pregnancy cycle.

In summary, we have explored the role of immune factors in the pathogenesis of preterm and term preeclampsia, identifying four biomarkers that are concurrently involved in both subtypes of preeclampsia and exhibit high clinical diagnostic value. We have assessed their potential biological functions and diagnostic value for preeclampsia. The expression of the genes of interest was preliminarily validated through RT-PCR. The findings of this study provide guidance for future mechanistic research and offer new insights into the diagnosis and immunotherapy of preeclampsia.

### Electronic supplementary material

Below is the link to the electronic supplementary material.

Supplementary Material 1

Supplementary Material 2

Supplementary Material 3

Supplementary Material 4

## Data Availability

The GSE75010 mRNA profiles were downloaded from the GEO database (https://www.ncbi.nlm.nih.gov/geo/)

## Abbreviations

WGCNA: Weighted gene co-expression network analysis
GSEA: Gene set enrichment analysis
PIK3CB: Phosphatidylinositol-4,5-bisphosphate 3-kinase catalytic subunit beta
CXCR6: C-X-C Motif Chemokine Receptor 6
IL1RAP: Interleukin 1 Receptor Accessory Protein
OSMR: Oncostatin M Receptor
PLGF: Placental Growth Factor
