# Supplementary figures and images for: Exploration of the molecular characteristics and potential clinical significance of shared immune-related genes between preterm preeclampsia and term preeclampsia
Source: BMC Pregnancy Childbirth. 2024 Aug 15;24:543. doi: 10.1186/s12884-024-06526-8 (PMC11328443; doi:10.1186/s12884-024-06526-8)

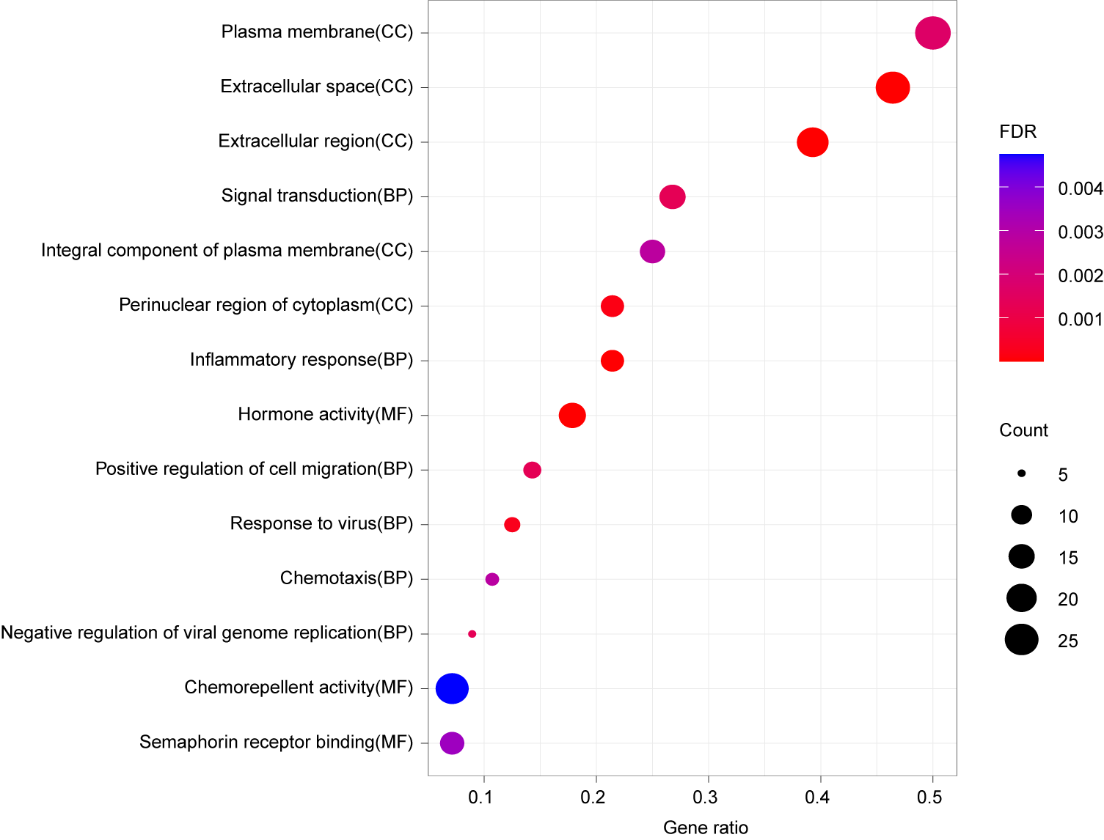


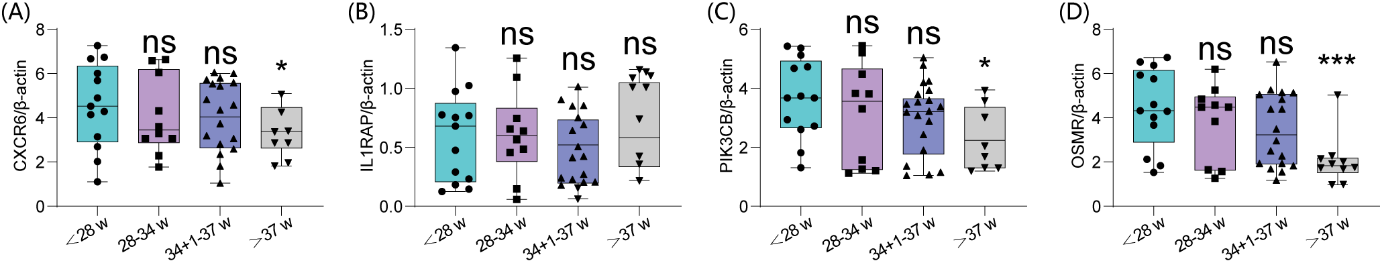

Supplement: Supplementary file 1 — Supplementary Material 1 [file 12884_2024_6526_MOESM1_ESM.docx]
